# Proprotein convertase subtilisin/kexin type 9 is a psoriasis-susceptibility locus that is negatively related to *IL36G*

**DOI:** 10.1172/jci.insight.141193

**Published:** 2022-08-22

**Authors:** Alexander Merleev, Antonio Ji-Xu, Atrin Toussi, Lam C. Tsoi, Stephanie T. Le, Guillaume Luxardi, Xianying Xing, Rachael Wasikowski, William Liakos, Marie-Charlotte Brüggen, James T. Elder, Iannis E. Adamopoulos, Yoshihiro Izumiya, Annie R. Leal, Qinyuan Li, Nikolay Y. Kuzminykh, Amanda Kirane, Alina I. Marusina, Johann E. Gudjonsson, Emanual Maverakis

**Affiliations:** 1 Department of Dermatology, University of California, Davis (UCD), Sacramento, California, USA.; 2 Department of Dermatology, University of Michigan, Ann Arbor, Michigan, USA.; 3 Department of Dermatology, University Hospital Zurich, Zurich, Switzerland.; 4 Swiss Institute for Allergy Research, Davos, Switzerland.; 5 Division of Rheumatology and Clinical Immunology, Harvard Medical School, Beth Israel Medical Deaconess Center, Boston, Massachusetts, USA.; 6 Department of Biochemistry and Molecular Medicine, UCD, Sacramento, California, USA.; 7 Institute of Biochemical Physics, Russian Academy of Science, Moscow, Russia.; 8 Department of Surgery, UCD, Sacramento, California, USA.

**Keywords:** Autoimmunity, Dermatology, Clinical practice, Skin

## Abstract

Proprotein convertase subtilisin/kexin type-9 (PCSK9) is a posttranslational regulator of the LDL receptor (LDLR). Recent studies have proposed a role for PCSK9 in regulating immune responses. Using RNA-Seq–based variant discovery, we identified a possible psoriasis-susceptibility locus at 1p32.3, located within *PCSK9* (rs662145 C > T). This finding was verified in independently acquired genomic and RNA-Seq data sets. Single-cell RNA-Seq (scRNA-Seq) identified keratinocytes as the primary source of *PCSK9* in human skin. *PCSK9* expression, however, was not uniform across keratinocyte subpopulations. scRNA-Seq and IHC demonstrated an epidermal gradient of PCSK9, with expression being highest in basal and early spinous layer keratinocytes and lowest in granular layer keratinocytes. *IL36G* expression followed the opposite pattern, with expression highest in granular layer keratinocytes. *PCSK9* siRNA knockdown experiments confirmed this inverse relationship between *PCSK9* and *IL36G* expression. Other immune genes were also linked to *PCSK9* expression, including *IL27RA*, *IL1RL1*, *ISG20*, and *STX3*. In both cultured keratinocytes and nonlesional human skin, homozygosity for *PCSK9* SNP rs662145 C > T was associated with lower *PCSK9* expression and higher *IL36G* expression, when compared with heterozygous skin or cell lines. Together, these results support *PCSK9* as a psoriasis-susceptibility locus and establish a putative link between PCSK9 and inflammatory cytokine expression.

## Introduction

Over the past decade, GWAS have identified over 60 psoriasis-susceptibility loci (1). However, psoriasis has an estimated heritability of 80%; thus, association studies have likely only captured a fraction of this heritability. Contrariwise, there are thousands of differentially expressed genes (DEGs) in psoriatic skin (2), but only a small fraction are known to contribute to psoriasis pathophysiology. The vast majority of DEGs remain unexplored in psoriasis. Several of these unexplored psoriasis DEGs are being actively pursued as therapeutic targets for other diseases, making the list of ready-to-administer drugs with potential efficacy in psoriasis expansive. Thus, increasing our knowledge of genetic predisposition to psoriasis may identify additional therapeutic targets.

Recently, expression of PCSK9, a posttranslational regulator of the LDL receptor (LDLR) that leads to LDLR internalization and degradation (3), has been shown to be altered in animal models of psoriasis and in the serum and skin of patients with psoriasis (4, 5). Furthermore, the *PCSK9* inhibitor evolocumab, which is approved for the treatment of hypercholesterolemia, has been associated with the development of various inflammatory skin conditions including psoriasis (6, 7). Studies have also demonstrated a role of PCSK9 in the regulation of apoptosis and proinflammatory cytokine secretion from macrophages (8). Although *PCSK9* SNPs (e.g., rs662145 C > T) have been linked to cardiovascular disease and Alzheimer’s disease (9, 10), their association with psoriasis remains unknown. Similarly, the expression patterns and role of PCSK9 in human keratinocytes in healthy individuals and patients with psoriasis are uninvestigated.

Here, we explore *PCSK9* as a possible psoriasis-susceptibility locus. We show that an SNP at the 3′ untranslated region of *PCSK9* (rs662145 C > T) predisposed individuals to psoriasis, a finding that was verified in independently acquired psoriasis RNA-Seq and genomic data sets. Using single-cell RNA-Seq (scRNA-Seq), keratinocytes were identified as the predominant cellular source of *PCSK9* in human skin. Analysis of *PCSK9* in cultured primary keratinocytes revealed a strong negative correlation between *PSCK9* and *IL36G* and *IL36B*, a relationship further supported by IHC data. Specifically, an epidermal gradient of PCSK9 was detected with expression highest in basal and early spinous layer keratinocytes and lowest in granular layer keratinocytes. In contrast, the gradient was reversed for IL-36, which was predominantly expressed in granular layer keratinocytes. *PCSK9* siRNA knockdown in immortalized keratinocytes confirmed the inverse relationship between PCSK9 and *IL36*. Finally, we found that *IL36* expression was increased in individuals homozygous for the *PCSK9* (rs662145 C > T) variant when compared with their heterozygous counterparts. Together these results link *PCSK9* to psoriasis and inflammatory cytokine production in human skin.

## Results

### PCSK9 is a possible psoriasis-susceptibility locus.

**Figure 1:**
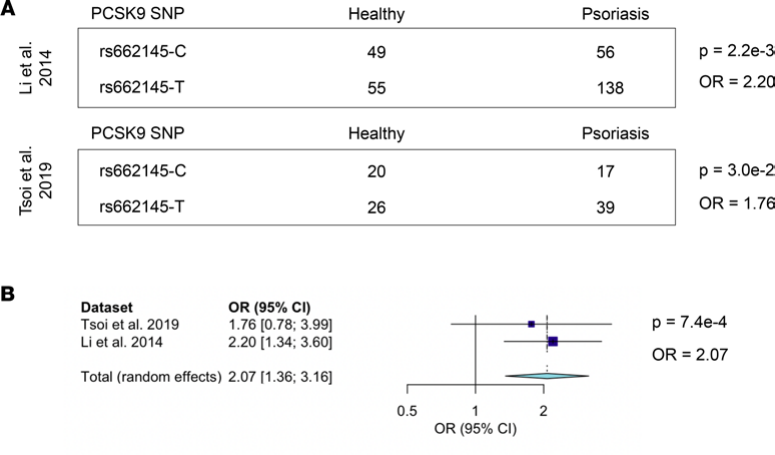
RNA-Seq variant calling identified a psoriasis-associated SNP in 3′ UTR of the *PCSK9* gene. (**A**) SNP calling was performed on 2 separate psoriasis RNA-Seq data sets (12, 14), and ORs were calculated using the allele counting method. Fisher’s exact test was performed to calculate *P* values. Names of analyzed data sets are shown on the left with OR and *P* values on the right. The presence of SNP rs662145 C > T was associated with increased psoriasis risk. SNP rs662145-C constitutes the reference and minor allele, and SNP rs662145-T constitutes the alternative and major allele. (**B**) Meta-analysis of both RNA-Seq data sets using a random-effects model.

**Figure 2:**
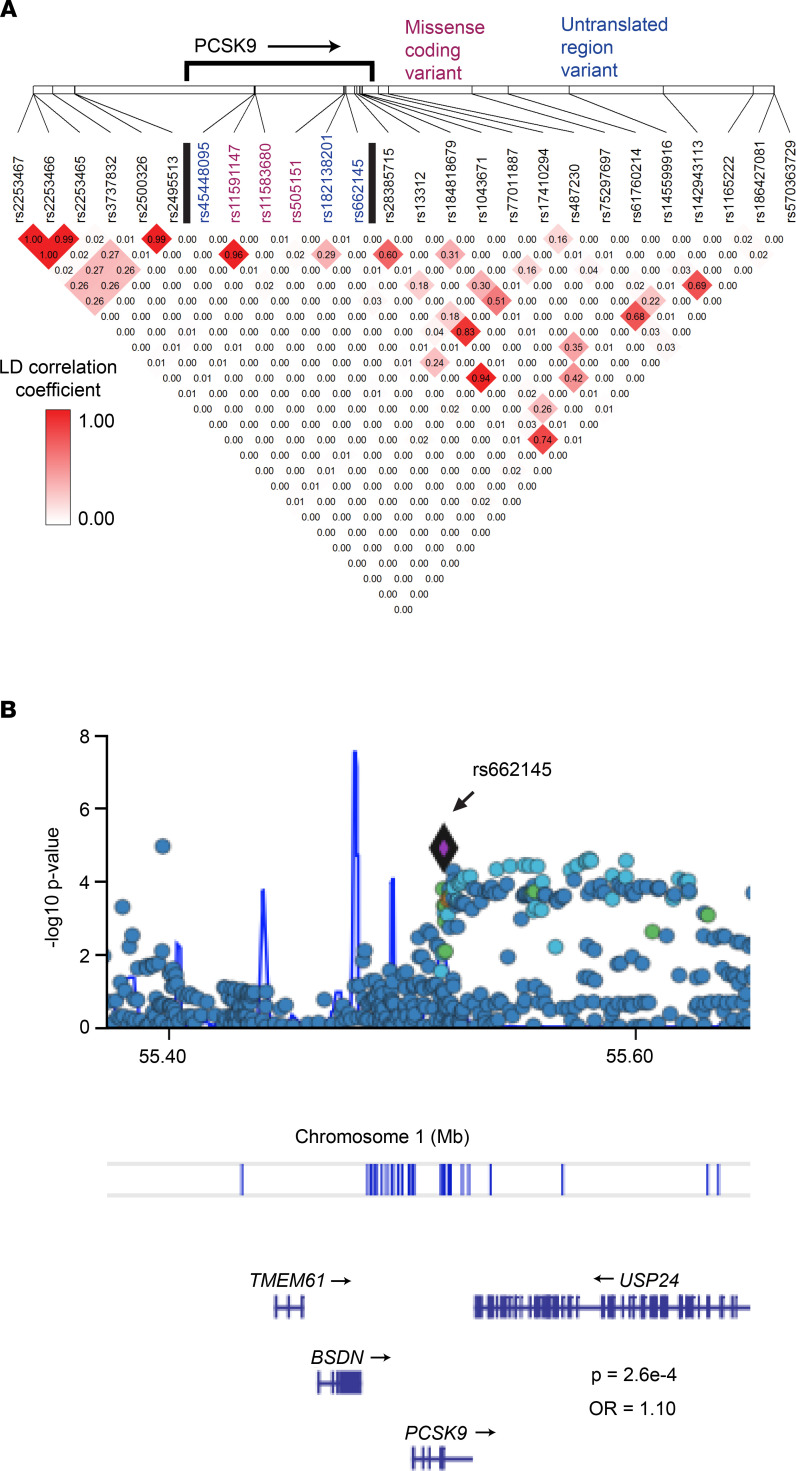
Genomic data confirmed a significant linkage between *PCSK9* SNP rs662145 C > T and psoriasis. (**A**) *PCSK9* SNP linkage disequilibrium estimated from a psoriasis RNA-Seq data set (12) (see data set details in Supplemental Table 1). (**B**) Analysis of GWAS data from 2590 cases of psoriasis and 1720 controls (13) revealed a significant linkage between *PCSK9* rs662145 C > T and psoriasis (*P* = 2.6 × 10^–4^).

Given that patients on *PCSK9* inhibitors have been reported to develop various inflammatory skin eruptions (6, 7), we used RNA-Seq–based genetic variant discovery to mine the largest published psoriasis transcriptome data set (11) for SNPs of *PCSK9*. This analysis identified 13 *PCSK9* SNPs. When compared with healthy controls, SNP rs662145 C > T was more prevalent among patients with psoriasis, a finding that remained significant after adjusting for multiple testing (OR = 2.20, *P* = 2.2 × 10^–3^). This finding was then confirmed in a second published independent psoriasis data set (OR = 1.76, *P* = 3.0 × 10^–2^) (Figure 1, A and B). A meta-analysis of these 2 data sets yielded a final combined OR of 2.07 (*P* = 6.5 × 10^–3^) (Figure 1B, and Supplemental Table 1; supplemental material available online with this article; https://doi.org/10.1172/jci.insight.141193DS1). The RNA-Seq–based genetic variant discovery method used to establish this linkage was then validated by calculating the linkage disequilibrium of rs662145 C > T with other nearby alleles (12), comparing the RNA-based results to those obtained from the 1000 Genomes Project data set as the gold standard. This analysis revealed a similar pattern of linkage disequilibrium for rs662145 C > T regardless of the data type used (Figure 2A, and Supplemental Figure 1). To further confirm the association between *PCSK9* SNP rs662145 C > T and psoriasis, we next examined the 1p32.3 genomic region in 2590 patients with psoriasis and 1720 controls, using data from previously published and recently acquired data sets (13). Similar to the results obtained from our variant discovery method, the genomic data revealed a significant linkage between *PCSK9* rs662145 C > T and psoriasis (OR = 1.10, *P* = 2.6 × 10^–4^; Figure 2B, purple diamond). Thus, in multiple independent data sets, *PCSK9* rs662145 C > T was associated with an increased risk of psoriasis.

### Psoriasis-linked PCSK9 variant rs662145 C > T is associated with altered PCSK9 and IL36 expression.

**Figure 3:**
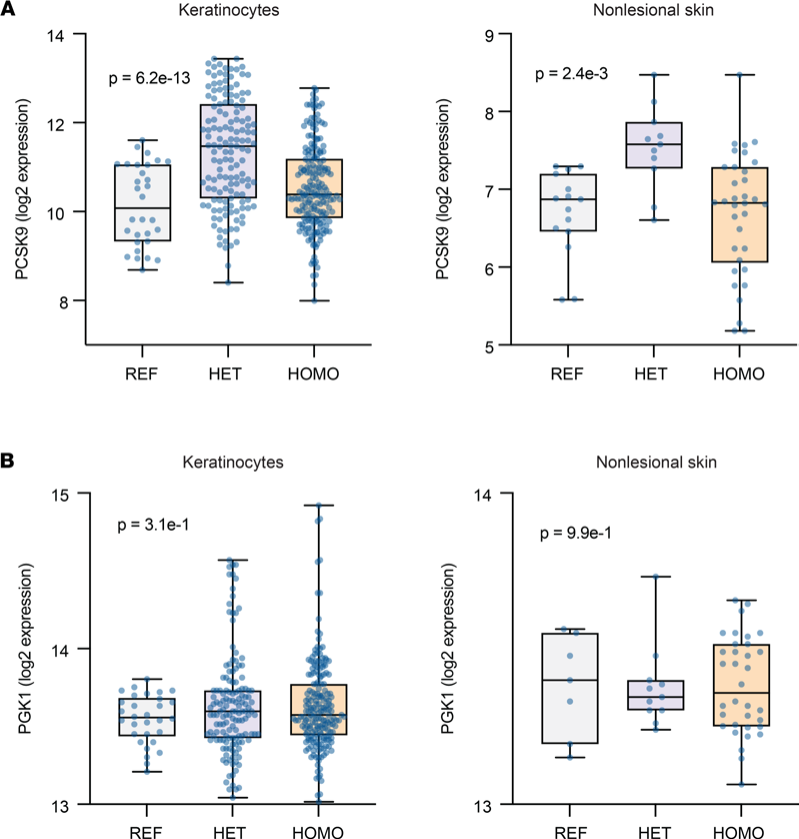
*PCSK9* SNP rs662145 C > T is associated with altered expression of *PCSK9* in cultured keratinocytes and nonlesional skin. (**A**) In cultured keratinocyte cell lines and nonlesional skin, *PCSK9* SNP rs662145 C > T HOMO phenotypes expressed lower levels of *PCSK9* compared with *PCSK9* SNP rs662145 C > T HET phenotypes. Box-and-whisker plots show normalized *PCSK9* expression (log transformed reads on *y* axis). Each dot represents 1 keratinocyte line or skin sample. Differential gene expression was calculated using DESeq2. FDR-adjusted *P* values are displayed on each plot. (**B**) In cultured keratinocyte cell lines and nonlesional skin, there was no difference in the expression of *PGK1,* a housekeeping gene, between the reference allele and *PCSK9* SNP rs662145 C > T HET and HOMO phenotypes. HOMO, homozygous; HET, heterozygous.

After identifying PCSK9 as a possible psoriasis-susceptibility locus, we next sought to determine how the psoriasis-associated *PCSK9* variant, rs662145 C > T, may predispose individuals to psoriasis. We first parsed 2 RNA-Seq data sets — a newly generated data set derived from primary human keratinocytes isolated from healthy individuals without psoriasis and a data set derived from nonlesional skin that we previously generated (14) — to characterize the effect of SNP rs662145 on *PCSK9* expression. Box-and-whisker plots of *PCSK9* expression were constructed for each data set (Figure 3, A and B). Within each data set, samples were parsed into 3 groups based on the *PCSK9* allele they expressed — i.e., homozygous for the reference allele (SNP rs662145-C), heterozygous for the alternative allele (SNP rs662145-T), and homozygous for the alternative allele. This analysis revealed that when compared with their homozygous counterparts, primary human healthy keratinocytes heterozygous for *PCSK9* SNP rs662145 C > T exhibited increased expression of *PCSK9* (*P* = 6.2 × 10^–13^; Figure 3A). The same pattern of *PCSK9* expression was also observed in nonlesional human skin (*P* = 2.4 × 10^–3^; Figure 3A).

**Figure 4:**
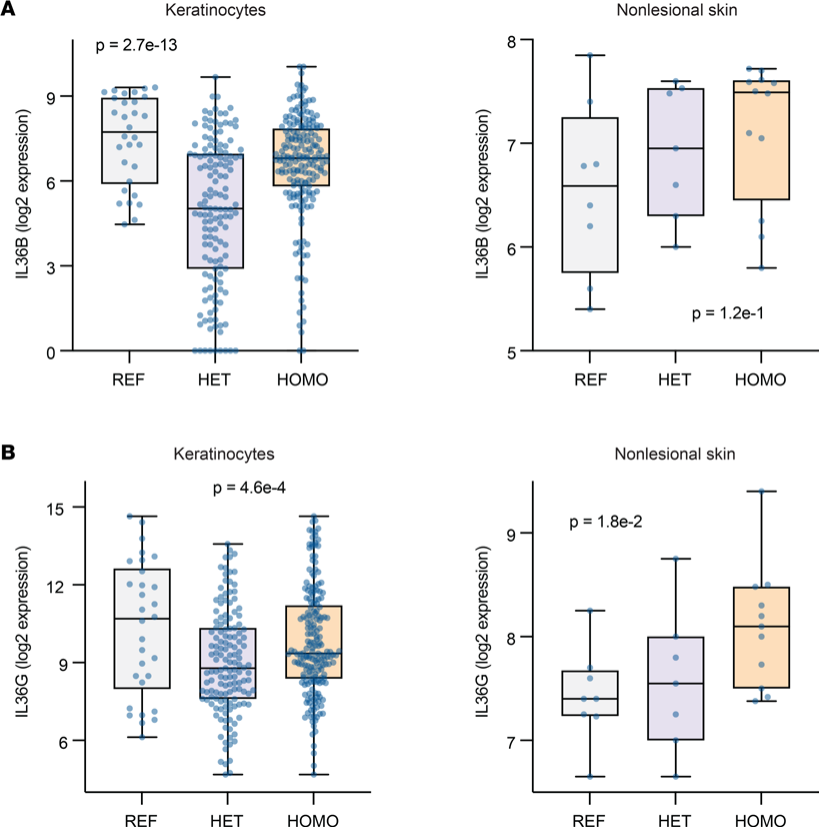
*PCSK9* SNP rs662145 C > T is associated with altered expression of *IL36* in cultured keratinocytes and nonlesional skin. (**A**) In cultured keratinocyte cell lines and nonlesional skin, *PCSK9* SNP rs662145 C > T HOMO phenotypes expressed higher levels of *IL36B* compared with *PCSK9* SNP rs662145 C > T HET phenotypes. Box-and-whisker plots show normalized *PCSK9* expression (log transformed reads on *y* axis). Each dot represents 1 keratinocyte line or skin sample. Differential gene expression was calculated using DESeq2. FDR-adjusted *P* values are displayed on each plot. (**B**) In cultured keratinocyte cell lines and nonlesional skin, *PCSK9* SNP rs662145 C > T HOMO phenotypes expressed higher levels of *IL36G* compared with *PCSK9* SNP rs662145 C > T HET phenotypes. HOMO, homozygous; HET, heterozygous.

We next aimed to establish if *PCSK9* expression was linked to any well-known mediator of psoriasis. Examining *PCSK9* expression in cultured keratinocytes revealed that SNP rs662145 C > T was associated with differential expression of *IL36*, an IL-1 family member and psoriasis-defining cytokine (15, 16). Comparing *IL36* expression in primary human keratinocytes homozygous and heterozygous for *PCSK9* SNP rs662145 C > T revealed that homozygosity for rs662145 C > T was associated with lower expression levels of *PCSK9* (Figure 3A) and higher levels of *IL36B* and *IL36G* (Figure 4, A and B). This same expression pattern was also observed in nonlesional human skin — i.e., homozygosity for *PCSK9* SNP rs662145 C > T was associated with lower *PCSK9* expression (Figure 3A) and higher *IL36G* expression (Figure 4B) when compared with heterozygosity for *PCSK9* SNP rs662145 C > T.

However, the relationship between homozygosity for the *PCSK9* reference allele and *IL36* expression diverged between keratinocyte cultures and nonlesional skin. In human keratinocyte cultures, homozygosity for the *PCSK9* reference allele was associated with lower expression of *PCSK9* (Figure 3A) and higher expression of *IL36B* and *IL36G* (Figure 4, A and B). In contrast, in nonlesional human skin, *IL36G* expression was lower in individuals homozygous for the *PCSK9* reference allele, with an apparent dose-response relationship between the *PCSK9* rs662145 C > T variant and baseline *IL36G* expression (Figure 4B). Thus, while homozygosity for the psoriasis-linked *PCSK9* variant rs662145 C > T was consistently associated with lower *PCSK9* expression and higher *IL36G* expression, homozygosity for the *PCSK9* reference allele appeared to yield different results in vitro and in vivo.

### PCSK9 is negatively related to keratinocyte-derived IL36.

**Figure 5:**
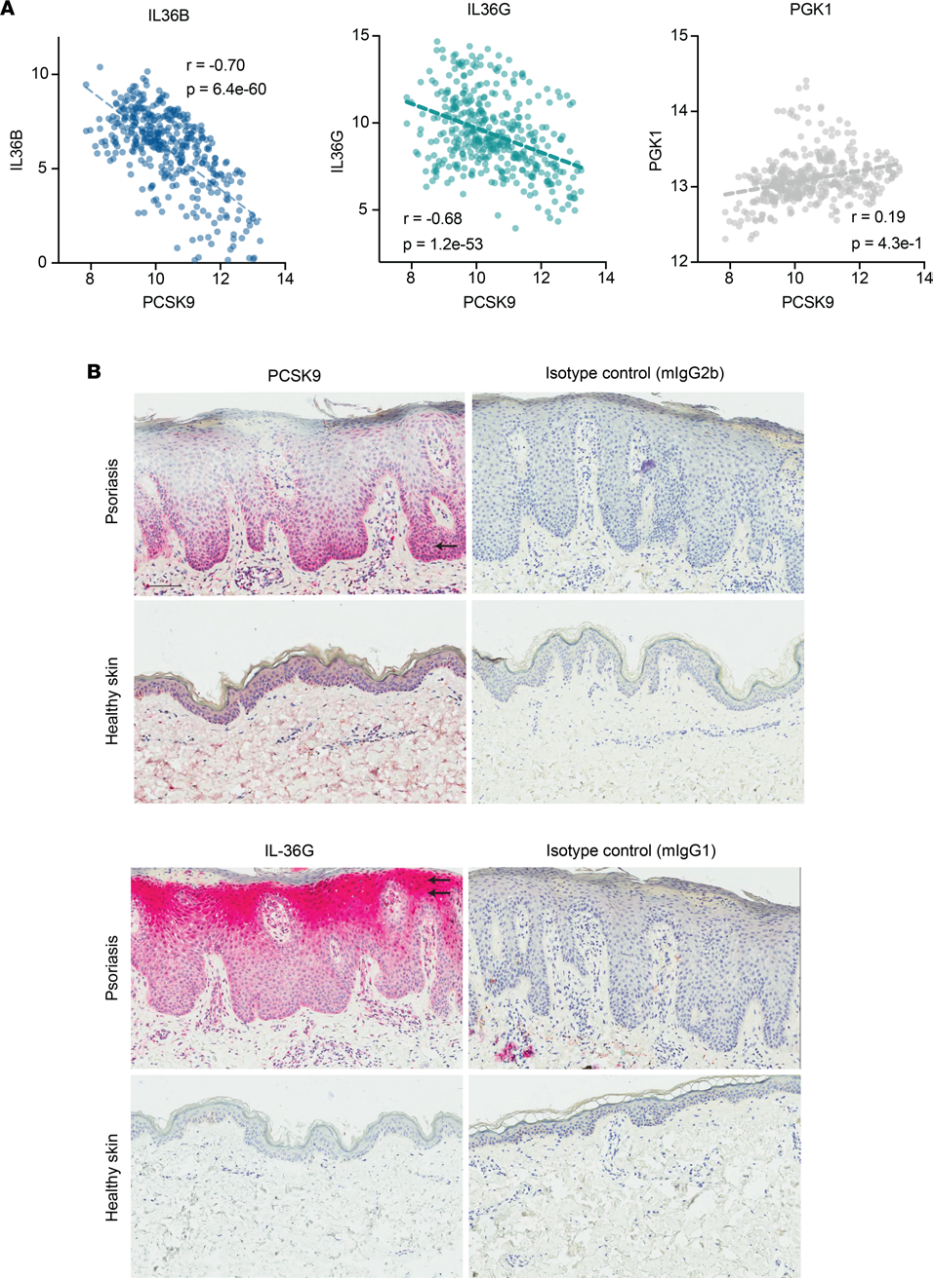
*PCSK9* expression negatively correlates with *IL36B* and *IL36G* expression in keratinocytes and skin. (**A**) In cultured keratinocyte cell lines, *PCSK9* expression negatively correlated with *IL36B* and *IL36G* expression. *PCSK9* expression did not correlate with *PGK1* expression, a housekeeping gene. In these plots, each dot represents an in vitro cultured keratinocyte cell line under a different culture condition (control, IL-4, IL-13, IL-17A, IFN-α, IFN-γ, TNF-α, IL-4 and IL-13, IL-17A and IFN-γ, IL-17A, and TNF-α). Normalized log_2_ transformed reads for each gene are plotted on the *x* axis and *y* axis. Pearson’s correlation coefficients and *P* values are displayed on each plot. (**B**) Epidermal expression of *PCSK9* and *IL36G*. Representative pictures of an IHC staining for *PCSK9* and *IL36G* as well as the matching isotype controls in lesional skin of a patient with psoriasis (upper row) and healthy control nonlesional skin (lower row). The single arrow points to the basal layer. Double arrows point to the granular layer. Scale bar: 100 μm.

Having established that expression of *PCSK9* rs662145 C > T variant was associated with increased expression of keratinocyte-derived *IL36*, we next aimed to elucidate the relationship between *PCSK9* and *IL36*. We cultured healthy primary keratinocytes and found that *PCSK9* negatively correlated with *IL36B* and *IL36G* expression (*r* = –0.70, *P* = 6.4 × 10^–60^; and *r* = –0.68, *P* = 1.2 × 10^–53^, respectively; Figure 5A). No significant correlation was noted between *PCSK9* and *PGK1*, a housekeeping gene (Figure 5A). We then performed IHC staining, which also highlighted this negative relationship between PCSK9 and IL-36γ expression in psoriatic skin — i.e., where epidermal expression of PCSK9 was high, IL-36γ expression was low (Figure 5B). PCSK9 expression was most prominent in the basal and early spinous layers of the epidermis, whereas IL-36γ expression was highest in the granular layer (Figure 5B).

**Figure 6:**
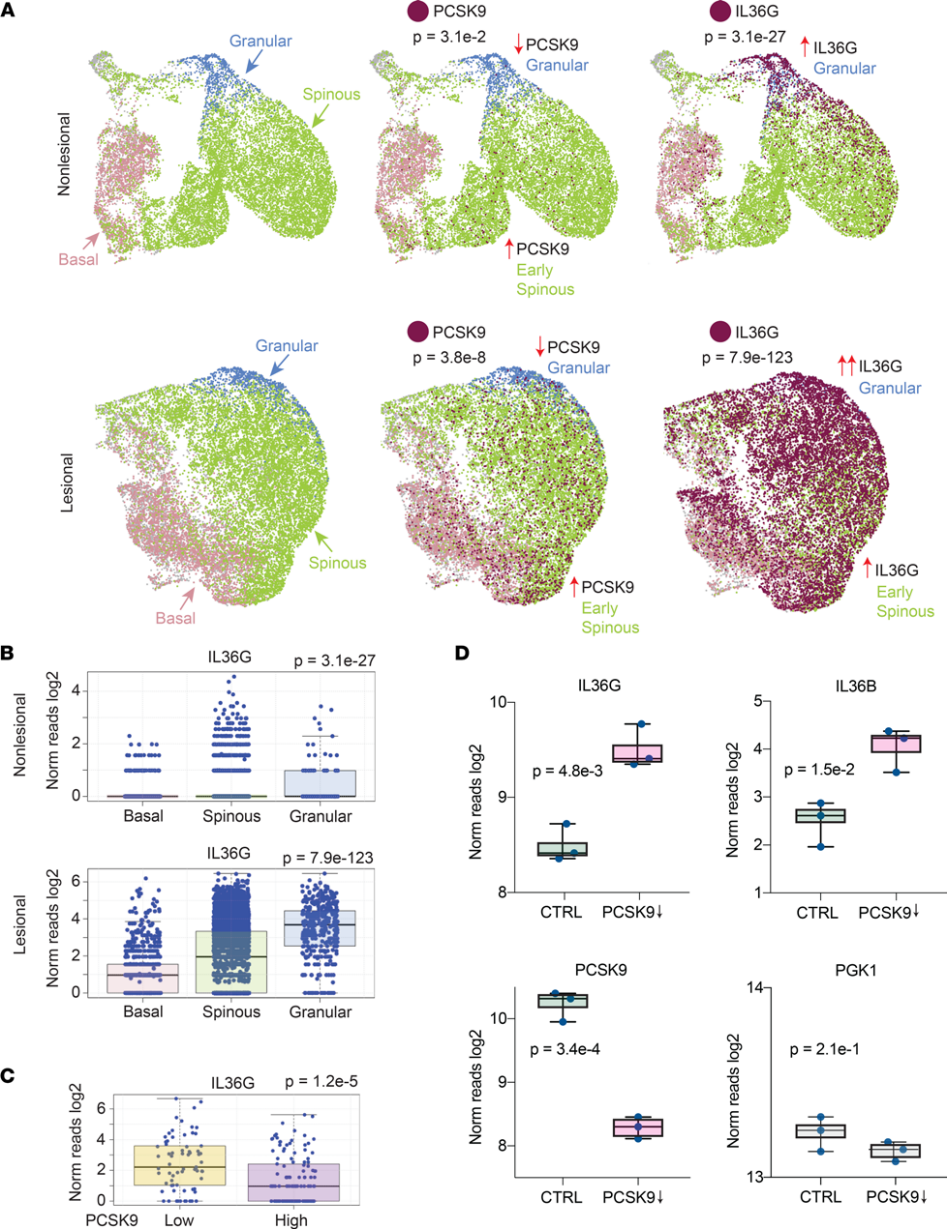
*PCSK9* expression is negatively and directly related to *IL36B* and *IL36G* expression. (**A**) Single-cell sequencing of psoriatic nonlesional and lesional skin (*n* = 9). The UMAP method was used to create 2-dimensional representation of the resulting data. Keratinocyte populations were identified by the expression levels of established keratinocyte markers (red, basal layer keratinocytes, *DST* high; green, spinous layer keratinocytes, *KRT5* low and *KLK7* low; and blue, granular layer keratinocytes, *KLK7* high). *PCSK9-* and *IL36G*-expressing cells are depicted in maroon. (**B**) Expression of *IL36G* in individual basal, spinous, and granular layer keratinocytes shown as box-and-whisker plots of log_2_ transformed gene expression. *P* values were calculated for each data set using 1-way ANOVA. (**C**) *PCSK9*-positive keratinocytes were parsed into 2 groups, *PCSK9*-high and *PCSK9*-low (*x* axis). Box-and-whisker plots of indicated intracellularly expressed genes are plotted on the *y* axis (log_2_ reads). *P* values were calculated using Student’s *t* test. (**D**) Box plots showing the effects of in vitro siRNA knockdown of *PCSK9* in keratinocyte cell lines on *IL36B* and *IL36G*. *PCSK9* (positive control) and *PGK1* (negative control) expression is also shown for scrambled siRNA transfected and PCSK9 siRNA transfected cultures. Each dot represents an independently cultured and independently transfected HaCaT keratinocyte cell line (*n* = 3). *P* values were calculated with Student’s *t* test.

The same expression pattern was also noted in our scRNA-Seq data of psoriatic nonlesional and lesional skin (*n* = 9) (Figure 6, A–C). The Uniform Manifold Approximation and Projection (UMAP) method was used to create 2-dimensional representations of the resulting data. Basal, spinous, and granular layer keratinocyte populations were identified using validated keratinocyte-expressed genes. scRNA-Seq also revealed that, in human skin, the vast majority of *PCSK9* expression could be attributed to keratinocytes (Supplemental Figure 2). Analysis of gene expression within each keratinocyte subpopulation demonstrated a gradient of *PCSK9* expression with expression being highest in basal and early spinous layer keratinocytes and lowest in granular layer keratinocytes (psoriatic nonlesional skin, *P* = 3.1 × 10^–2^; psoriatic lesional skin, *P* = 3.8 × 10^–8^; Figure 6A). In contrast, *IL36G* expression was highest in granular layer keratinocytes (psoriatic nonlesional skin, *P* = 3.1 × 10^–27^; psoriatic lesional skin, *P* = 7.9 × 10^–123^; Figure 6, A and B). Furthermore, when keratinocytes were parsed into *PCSK9* high and low expressors, *IL36G* expression was significantly higher in the *PCSK9* low-expressing keratinocytes (*P* = 1.2 × 10^–5^; Figure 6C).

To further verify this negative relationship between *PCSK9* and *IL36*, we used *PCSK9* siRNA to knock down *PCSK9* expression in an *IL36*-expressing HaCaT human keratinocyte cell line. The siRNA knockdown experiment was performed in 3 independently cultured HaCaT cell lines, and gene expression was assessed by RNA-Seq. In comparison to control siRNA knockdown cell cultures, *PCSK9* knockdown cultures expressed significantly more *IL36B* and *IL36G* (*P* = 1.5 × 10^–2^ and *P* = 4.8 × 10^–3^, respectively; Figure 6D), which confirmed the inverse relationship between *PCSK9* and *IL36*. No significant change in gene expression was noted for *PGK1*, a housekeeping gene.

### PCSK9 is linked to other potential mediators of psoriasis.

**Figure 7:**
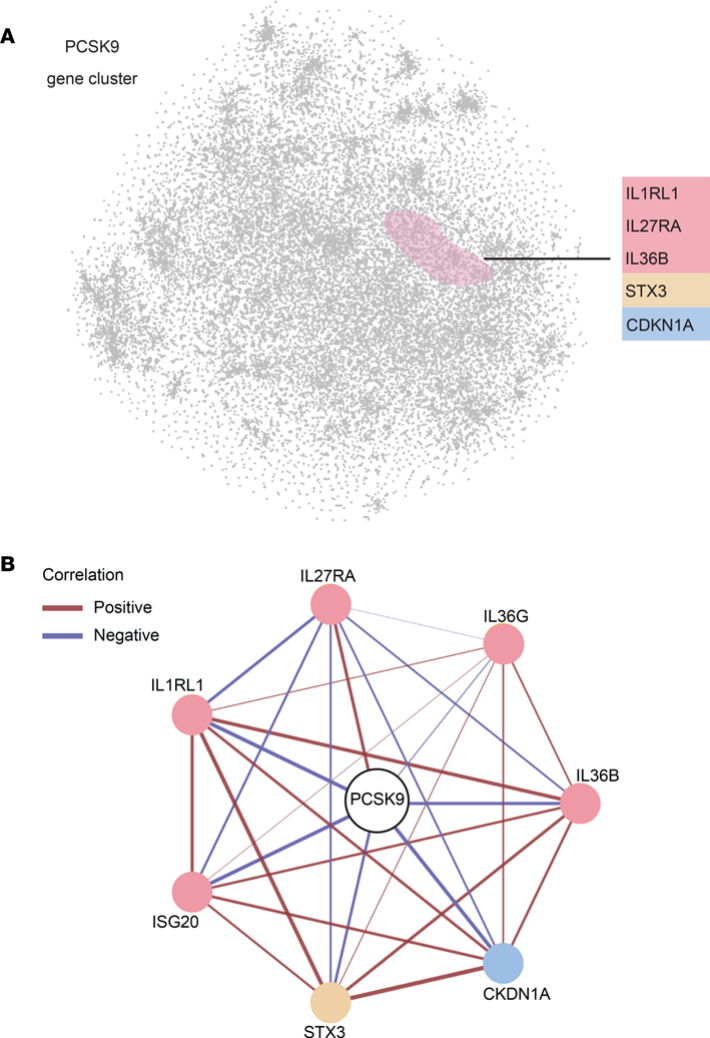
*PCSK9* expression clusters with inflammatory mediators of psoriasis. (**A**) A 2-dimensional plot of the keratinocyte transcriptome was constructed using a nonlinear dimensionality reduction strategy, the t-SNE method. Each point represents a gene and the distance between the points is inversely related to how well the genes correlate with one another. Within this plot, *PCSK9* clusters with various genes of interest, shown on the right of the plot. (**B**) *PCSK9* coexpression network with each circle representing a different gene and the lines connecting each circle representing the strength (thickness of line) and direction (red = positive, blue = negative) of each correlation.

We then investigated whether *PCSK9* is linked to other potential inflammatory mediators of psoriasis in addition to *IL36B* and *IL36G*. To further assess how *PCSK9* expression was associated with other keratinocyte-expressed psoriasis-relevant genes, we created a 2-dimensional plot of the keratinocyte transcriptome using the t-Distributed Stochastic Neighbor Embedding (t-SNE) dimensionality reduction method. In this plot, each data point represents a different gene expressed in keratinocytes and the distance between any 2 points represents how strongly the genes correlate with each other in their expression. Within this representation of the keratinocyte transcriptome, PCSK9 clustered with a variety of genes related to cytokines (*IL1RL1*, *IL27RA*, and *IL36B*), cytokine secretion (*STX3*), and keratinocyte proliferation (*CDKN1A*) (Figure 7A). To visualize these relationships, *PCSK9* gene coexpression network analysis was performed, which highlighted the different relationships between *PCSK9* and the various keratinocyte-expressed mediators of inflammation (Figure 7B).

**Figure 8:**
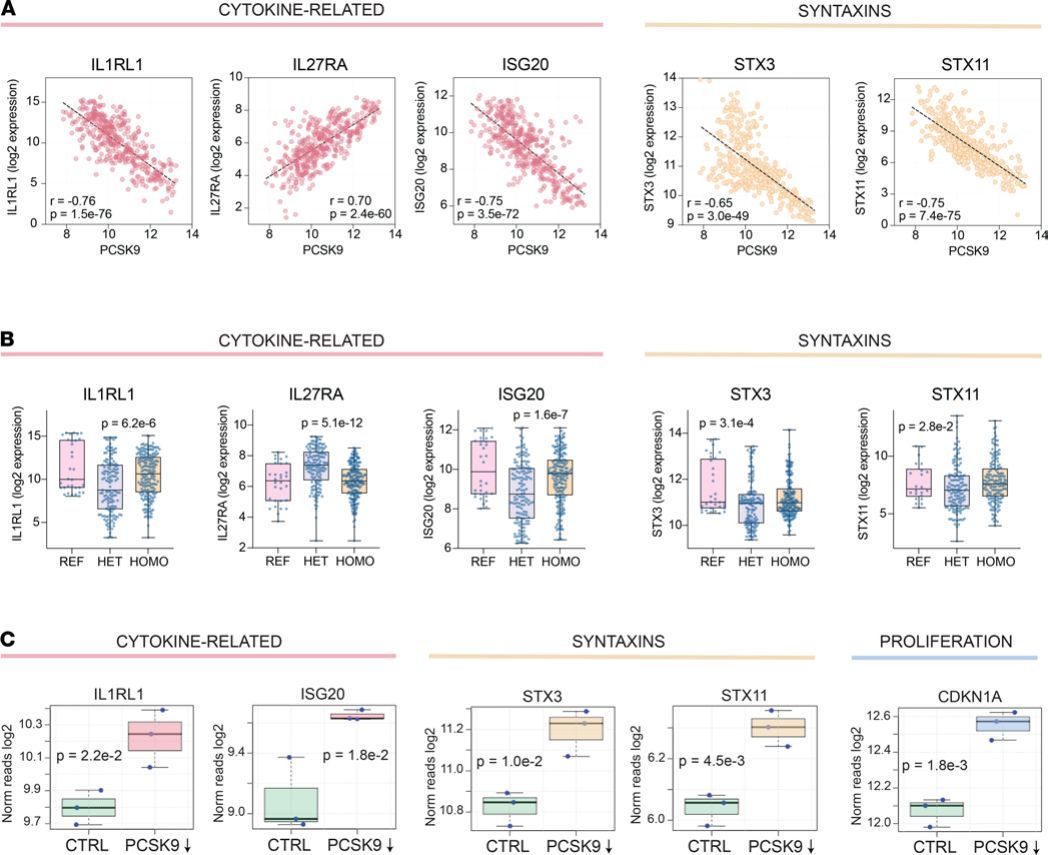
*PCSK9* expression directly correlates with inflammatory mediators of psoriasis. (**A**) Individual scatter plots showing correlations between *PCSK9* and *IL1RL1*, *IL27RA*, *ISG20*, *STX3*, and *STX11* in cultured keratinocytes. In these plots, each dot represents an in vitro cultured keratinocyte cell line under a different culture condition (control, IL-4, IL-13, IL-17A, IFN-α, IFN-γ, TNF-α, IL-4 and IL-13, IL-17A and IFN-γ, IL-17A, and TNF-α). *PCSK9* expression is shown on the *x* axis with each plot depicting a different gene on the *y* axis. (**B**) Box-and-whisker plots showing expression of genes of interest in cultured keratinocytes displaying the REF allele or rs662145 C > T variant *PCSK9* allele. Differential gene expression was calculated using DESeq2. REF, reference; HET, heterozygous; HOMO, homozygous. (**C**) Box plots showing effects of in vitro siRNA knockdown of *PCSK9* in keratinocyte cell lines on expression of genes of interest. Each dot represents an independently cultured and independently transfected HaCaT keratinocyte cell line (*n* = 3). The *y* axis depicts log_2_ transformed normalized reads and the *x* axis compares control keratinocyte cell lines with *PCSK9* knockdown cell lines, with *P* values calculated via Student’s *t* test.

To determine how well *PCSK9* correlates with these and other keratinocyte-expressed genes, we investigated patterns of gene expression in our cultured keratinocyte cell lines. We found that the expression of *IL1RL1, IL27RA*, *ISG20,* *STX3,* and *STX11* appeared to be linked to *PCSK9* in keratinocytes. Specifically, scatter plots of gene expression revealed that the expression of *PCSK9* positively correlated with *IL27RA* (Figure 8A; *r* = 0.70, *P* = 2.4 × 10^–60^) and negatively correlated with *IL1RL1, ISG20,* *STX3,* and *STX11* (*r* = –0.76, *P* = 1.5 × 10^–76^; *r* = –0.75, *P* = 3.5 × 10^–72^; *r* = –0.65, *P* = 3.0 × 10^–49^; and *r* = –0.75, *P* = 7.4 × 10^–75^, respectively) (Figure 8A). *IL1RL1*, *IL27RA*, *ISG20*, *STX3*, and *STX11* were also differentially expressed in primary human keratinocyte cell lines heterozygous and homozygous for the *PCSK9* variant rs662145 C > T (*P* = 6.2 × 10^–6^, *P* = 5.1 × 10^–12^, *P* = 1.6 × 10^–7^, *P* = 3.1 × 10^–4^, and *P* = 2.8 × 10^–2^, respectively) (Figure 8B). Similar to the pattern noted for *PCSK9*, primary keratinocyte cell lines heterozygous for *PCSK9* SNP rs662145 C > T had increased expression of *IL27RA* (Figure 8B). In contrast, the opposite pattern was associated with *IL1RL1, ISG20, STX3*, and *STX11*, where expression was decreased in lines heterozygous for *PCSK9* SNP rs662145 C > T (Figure 8B). Finally, *PCSK9* siRNA knockdown resulted in increased expression of *IL1RL1*, *ISG20*, *STX3*, *STX11*, and *CDKN1A* (Figure 8C), confirming a direct negative correlative relationship between *PCSK9* and these genes.

## Discussion

The major finding of our study is the identification of *PCSK9* as a psoriasis-susceptibility locus (Figures 1 and 2). This was established by mining psoriasis and healthy control RNA-Seq data sets for differential occurrences of *PCSK9* variants. A meta-analysis across 2 independently acquired RNA data sets yielded a highly statistically significant final model for this association (*P* = 7.4 × 10^–4^; Figure 1B). Furthermore, *PCSK9* genotyping data from 2590 psoriasis cases and 1720 controls also associated the exact same SNP, rs662145 C > T, to the psoriasis phenotype (*P* = 2.6 × 10^–4^; Figure 2B). Thus, across multiple data sets (RNA and DNA), SNP rs662145 C > T was consistently associated with psoriasis. However, there are major differences between traditional genotyping and the RNA-Seq–based variant discovery method used here. First, the RNA-Seq–based variant discovery method examined only a fraction of the total *PCSK9* variants, those which resided in the transcribed region of the gene. Also, the relatively small sample size of the RNA-Seq data sets makes it difficult to estimate the OR of the association. In fact, the OR estimated via RNA-Seq–based variant discovery was higher than the OR identified by genotyping the *PCSK9* locus. However, as these are different techniques, we expected them to yield different ORs. For example, unlike traditional genotyping methods, the variant discovery method used allele counting to calculate the OR. Thus, further investigation is needed to accurately estimate the OR for this association, especially since heterozygosity and homozygosity for *PCSK9* SNP rs662145 C > T may display unexpected effects, as described below.

It is becoming increasingly apparent that many causal variants alter disease risk through regulatory effects on gene expression (17). GWAS have uncovered hundreds of genetic variants linked to human diseases (18), yet functional biomechanistic studies aimed at understanding these linkages have lagged far behind. In addition to identifying *PCSK9* as a psoriasis-susceptibility locus, we performed the initial studies to understand how the psoriasis-linked *PCSK9* variant, SNP rs662145 C > T, might predispose individuals to psoriasis. Here, we establish that SNP rs662145 C > T is associated with altered *PCSK9* expression in keratinocytes. This was determined by parsing the primary human keratinocyte RNA-Seq data sets into 3 groups (homozygous for the *PCSK9* reference allele, heterozygous for *PCSK9* SNP rs662145 C > T, and homozygous for *PCSK9* SNP rs662145 C > T). When compared with heterozygous keratinocyte cell lines, *PCSK9* SNP rs662145 C > T homozygous cell lines expressed low levels of *PCSK9* and high levels of *IL36* cytokines, a family of cytokines induced by IL-17A in autoimmunity and inflammatory skin conditions (19). IL-36β and IL-36γ are known to promote epidermal hyperplasia, hyperkeratosis, and cutaneous inflammation in psoriasis, and IL-36γ is thought to be a key driver of psoriasis pathogenesis (14, 20), especially pustular psoriasis (21). The negative correlative relationship between *PCSK9* and *IL36* expression is an important finding of our study. It was consistently observed across multiple experimental approaches, including IHC, RNA-Seq, scRNA-Seq, and *PCSK9* siRNA knockdown (Figures 5 and 6). This inverse relationship provides a plausible explanation as to how *PCSK9* SNP rs662145 C > T may predispose individuals to psoriasis — i.e., lowering PCSK9 expression in keratinocytes increases baseline cutaneous expression of IL-36, a psoriasis-driving cytokine. Although not explored here, other investigators have linked loss-of-function and gain-of-function *PCSK9* variants to disease (22).

The fact that keratinocyte cell lines homozygous for the PCSK9 reference allele also had low *PCSK9* expression and high *IL36* expression was difficult to reconcile. If *PCSK9* SNP rs662145 C > T predisposes individuals to psoriasis by increasing their baseline cutaneous expression of IL-36, one would expect that keratinocytes homozygosity for the *PCSK9* reference allele should have low *IL36* expression, as individuals homozygous for the *PCSK9* reference allele are not predisposed to psoriasis. Indeed, these in vitro results differed from those we observed in nonlesional human skin, where homozygosity for the *PCSK9* reference allele was associated with low *IL36G* expression. In fact, in nonlesional skin RNA-Seq data demonstrated a linear relationship between the dose of *PCSK9* SNP rs662145 C > T and the amount of baseline *IL36G* detected (Figure 4B). This finding is highly relevant because polymorphisms in *IL36* have been implicated in plaque psoriasis (23), and this cytokine has increasingly been identified as an important driver of other immune-mediated skin diseases, including pyoderma gangrenosum and hidradenitis suppurativa (24–29). Although the differences between cultured keratinocytes and human skin were not further explored here, it is important to note that PCSK9 can exist as a monomer, dimer, and trimer (30, 31). This may be a reason why our in vitro keratinocyte findings did not perfectly translate to in vivo settings. A major finding that was consistent across the in vitro and in vivo settings was the observation that *PCSK9* SNP rs662145 C > T homozygous individuals and cell lines express less *PCSK9* and more *IL36G* when compared with their heterozygous counterparts.

To date, reports regarding the effect of PCSK9 inhibition on skin inflammation have been conflicting. In an imiquimod mouse model of psoriasis-like dermatitis, suppressing PCSK9 with siRNA resulted in improvement of skin lesions (5). However, the effects of PCSK9 suppression in human skin remain poorly characterized. Two PCSK9 inhibitors, alirocumab and evolocumab, are now FDA-approved to reduce circulating LDL levels and lower all-cause mortality in patients with coronary artery disease (32). In patients on these inhibitors, case reports have documented various inflammatory skin eruptions, including psoriasis and atopic dermatitis (6, 7). The development of atopic dermatitis while on PCSK9 inhibition was accompanied by a high serum IgE (6). Although most commonly studied in atopic dermatitis, IgE is also elevated in patients with psoriasis (33), which may be due to the increased expression of IL-36 in this disease (14). Specifically, IL-36 has recently been linked to Ig isotype switching to IgE following cutaneous antigen exposure (34).

Thus, in addition to its well-characterized ability to bind to LDLR, PCSK9 appears to be strongly linked to mediators of innate and adaptive immunity. Although the mechanisms by which *PCSK9* variants may alter immune responses remain to be investigated, cholesterol-sulfate is a well-known and potent transcriptional regulator in keratinocytes (35). It is possible that the altered intracellular abundance of cholesterol-sulfate and phospholipids in *PCSK9* rs662145 C > T variant individuals results in the differential expression of proinflammatory cytokines (Supplemental Figure 3). Another possibility is that PCSK9 regulates removal of inflammatory lipids such as LPS via its effects on LDLR (36).

Here, we have focused on using alternative methods, including RNA-Seq–based genetic variant discovery and expression quantitative trait loci–like analysis strategies, to characterize immune responses from whole tissue RNA-Seq data sets and integrated scRNA-Seq to validate our findings (37, 38).

In summary, our study establishes *PCSK9* as a possible psoriasis-susceptibility locus and demonstrates its negative relationship with *IL36G* at the gene expression and protein levels. We also firmly establish an association between *PCSK9* SNP rs662145 C > T and *PCSK9* gene expression. Although gaps in our understanding of these relationships remain, our working hypothesis is that *PCSK9* SNP rs662145 C > T may predispose individuals to psoriasis by increasing baseline cutaneous expression of *IL36G*. Future mechanistic studies are needed to better elucidate these findings.

## Methods

### Human skin RNA-Seq data sets.

RNA-Seq FASTQ files of human normal and psoriatic lesional skin were downloaded from the NCBI Sequence Read Archive (http://www.ncbi.nlm.nih.gov/Traces/sra). A total of 4 data sets were used: 3 independent data sets (accession numbers: SRP165679, SRP026042, and SRP057087) and 1 data set comprised of 2 combined experimental data sets published by the same research group (accession numbers: SRP035988 and SRP050971) (11, 12, 14). The latter included 99 lesional psoriatic and 90 normal skin biopsies from people of European descent enrolled in the Southeast Michigan area. Patients with psoriasis who volunteered for a skin biopsy underwent a washout period of all immunosuppressive medications prior to the procedure.

### RNA-Seq.

Total RNA was extracted using the RNeasy Plus Mini Kit (Qiagen, catalog 74134). RNA concentrations were then quantified using a Qubit Fluorometer (Invitrogen), and RNA integrity was assessed using the Agilent TapeStation. Samples with an RNA integrity number greater than or equal to 8 were used for this study. Indexed libraries were constructed from 500 ng of total RNA using the TruSeq Stranded mRNA Sample Prep Kit (Illumina, catalog 20020594) following the manufacturer’s instruction. The quantity and quality of the libraries were also assessed by Qubit and Agilent 2100 Bioanalyzer, respectively. The average library size was 400 bp. Libraries’ molar concentrations were validated by quantitative PCR (qPCR) for library pooling. Sequencing was performed on the Illumina HiSeq 4000 platform using PE150 chemistry.

### Alignment and variant calling.

Raw sequencing data were received in FASTQ format. Alignment of sequencing reads to the reference genome (hg38) was performed using STAR v2.5.2 (39). Haplotype calling was performed using the Genome Analysis Toolkit (GATK v3.8) (40) and best practice guidelines to call variants from the RNA-Seq data. For the analysis, only known variants were analyzed. The National Center for Biotechnology Information (NCBI) database of genetic variation (dbSNP) release 146 was used as the reference for known SNPs. Gene annotation downloaded from the Ensembl website (http://www.ensembl.org/) was used for STAR mapping and the following read count evaluation. Gene expression level normalization and differential expression analysis was carried out using DESeq2 Bioconductor R package (version 1.6.3). This package provides statistics for determination of differential expression using a model based on the negative binomial distribution.

### Differential gene expression, correlation, and linkage.

Gene expression level normalization and differential expression analysis was carried out using DESeq2 Bioconductor R package (version 1.6.3) (41). This package provides statistics for determination of differential expression using a model based on the negative binomial distribution. Differential expression *P* values were corrected for multiple testing using the FDR method. Genes with FDR-adjusted *P* values less than 0.05 and fold change greater than 2 or less than 0.5 were considered differentially expressed. Correlation analyses of gene expressions were performed on read counts of each identified gene normalized with the DESeq2 package. Values were subsequently log transformed and winsorized when necessary. Pearson’s correlation coefficient (*r*) was calculated using the cor.test function in R (42). Spearman’s correlation coefficient (*r*_s_) was estimated by algorithm AS 89. When Pearson’s correlation coefficients were used, Cook’s distances were also calculated to ensure that there were no overly influential data points. The igraph software R package was used for the expression network visualization (43). Linkage disequilibrium among genes was calculated and visualized using R package gaston (44).

### 2D visualization of the keratinocyte transcriptome.

We computed gene pairwise distances using the formula, 1-r^2^, where r represents Pearson’s correlation coefficient. A visual representation of the gene coexpression network was then created using a dimensionality reduction technique, t-SNE, calculated with R package Rtsne (45).

### IHC.

FFPE skin blocks of 3 patients with psoriasis and 3 healthy controls were cut into 3 μm sections. The sections were first incubated at 70°C in an oven for 40 minutes to remove excess paraffin. With the help of Leica BOND RXm, they were deparaffinized and treated with EDTA buffer-based, pH 9.0 Epitope Retrieval Solution (ER2) for 30 minutes at 100°C. Sections were stained with an anti-PCSK9 (Thermo Fisher Scientific, catalog MA5-32843, mouse IgG2b, clone 2F1, 1:2000) and an anti–IL-36G (Abcam, catalog ab156783, mouse IgG1, clone OTI2F4, 1:500) using AP chromogen (BOND Polymer Refine Red Detection). The incubation time for both Abs was 30 minutes at room temperature. Sections were counterstained with hematoxylin.

### Meta-analysis.

Meta-analyses were completed using the R package metafor (46). A weighted random-effects model was used to estimate a summary effect size. To estimate between-study variance, a restricted maximum-likelihood estimator was applied. A weighted estimation with inverse-variance weights was used to fit the model.

### Single-cell sequencing.

Punch biopsies of 4 mm from patients with psoriasis and healthy controls were obtained (*n* = 9). Single cell suspensions were then prepared by mincing the biopsy tissue and digesting it in 0.2% Collagenase II (Life Technologies) and 0.2% Collagenase V (Sigma) in plain medium for 1 hour at 37°C and then strained through a 70 μM mesh. Single-cell sequencing libraries were then prepared using a 10X Genomics Chromium Controller. Libraries were sequenced on the Illumina NovaSeq 6000 sequencer to generate 151 bp paired-end reads. Data processing including quality control, read alignment, and gene quantification were conducted using 10X Genomics Cell Ranger v3.1 using their default parameters. Empty droplets and cells with unique molecular identifier (UMI) counts less than a calculated threshold based on the distribution of UMI counts per cell were removed. Data normalization and cell library size correction were performed using the median of ratios method implemented in the R package DESeq2. Clustered cells were mapped to corresponding cell types using the singleR R package and the Human Primary Cell Atlas as a reference data. Keratinocytes were then further subdivided into basal, spinous, and granular layer keratinocytes by matching their cell cluster gene signatures with cell type–specific markers (DST, KRT5, KRT10, and KLK7). Data have been deposited in the NCBI Gene Expression Omnibus (GEO) and are accessible through GEO Series accession numbers GSE121212 and GSE173706.

### Keratinocyte cell cultures.

The human immortalized keratinocyte cell line HaCaT (47) was cultured in DMEM (ATCC, catalog 30-2002) containing 1% Penicillin-Streptomycin Solution (ATCC, catalog 30-2300; penicillin G [10,000 U/mL] and streptomycin [10,000 μg/mL]) and 10% of Gibco FBS. Cells were maintained at 37°C in a humidified atmosphere containing 5% CO2 until they reached the exponential phase (2–3 weeks). Three passages were achieved before the experiments. Then, 3 × 10^5^ HaCaT cells were seeded in a 6-well flat-bottom tissue culture plate (Corning-Costar) in 2 mL of DMEM-supplemented per well. HaCaT cultures were treated with or without IL-4, IL-13, IL-17A, IFN-α, IFN-γ, TNF-α, IL-4 and IL-13, IL-17A and IFN-γ, IL-17A, and TNF-α). Expression analysis was performed using RNA-Seq as described above.

### siRNA-mediated knockdown of PCSK9.

For siRNA-mediated knockdown experiments, 3 parallel transfections were conducted using the SMARTpool siRNAs (mixture of 4 siRNAs), siRNA targeting PCSK9, and negative control siRNA, which were obtained from Dharmacon-Horizon Discovery. After overnight incubation, HaCaT cells (47) were transfected with 20 pmol of SMARTpool siRNAs using Lipofectamine RNAiMax Reagent (Thermo Fisher Scientific) as per the manufacturer’s instructions. After 48 hours, cells were washed with PBS and were harvested for RNA extraction using a mixture of Gibco Trypsin-EDTA (0.5%) and no phenol red (Thermo Fisher Scientific). Trypsin was neutralized with FBS and the cells were collected by centrifugation and resuspended in 100 μL of RNAlater. qPCR analysis was performed to test the efficiency of the transfection. Further expression analysis was performed using RNA-Seq as described above.

### Statistics.

All statistical analyses were performed using R (version 3.1.2) (42). In RNA-Seq analysis, fold changes were compared between groups using the Wald test implemented within the DESeq2 package (41). Correlation analysis was performed using the Pearson’s correlation coefficient, calculated in R (42) using cor.test function, which was also used to estimate *P* values for Pearson’s correlation coefficient using a 2-tailed Student’s *t* distribution test. Meta-analysis performed using R and *P* values were calculated using a Wald-type test for the coefficient of the meta-analysis model. Fisher’s exact test was used for testing association of SNPs with psoriasis. A 1-way ANOVA was used to test for association between SNP genotypes and gene expression levels. *P* values were adjusted for multiple testing using the Benjamini-Hochberg method, and adjusted *P* values less than 0.05 were considered statistically significant in all tests.

### Study approval.

Skin biopsies were obtained from volunteer patients following protocols approved by the University of Michigan Institutional Review Board. Written informed consent was obtained from all subjects.

## Author contributions

AM, AJX, AT, LCT, JEG, IEA, and EM wrote the manuscript. AM, AT, MCB, WL, QL, STL, LCT, GL, XX, RW, JTE, YI, ARL, NYK, AIM, AK, JEG, and EM acquired data. AM, AJX, NYK, LCT, JEG, and EM analyzed the data. All authors read, revised, and approved the final manuscript. The method used to assign the authorship order of the 2 co–first authors is as follows: AM was primarily responsible for data analysis, and AJX was primarily responsible for editing, review, revisions, and figures.

## Supplementary Material

Supplemental data

## Version 1 07/21/2022

In-Press Preview

## Version 2 08/22/2022

Electronic publication
