# Supplementary material for: Proprotein convertase subtilisin/kexin type 9 is a psoriasis-susceptibility locus that is negatively related to IL36G
Source: JCI Insight. 2022 Aug 22;7(16):e141193. doi: 10.1172/jci.insight.141193 (PMC9462487; doi:10.1172/jci.insight.141193)
Supplement: Supplemental data [file jciinsight-7-141193-s263.pdf]

## SUPPLEMENTAL MATERIAL

A

Gene: PCSK9 SNP: rs662145

| 1000 Genomes Project | Frequency | | Li et al. 2014 | Frequency - Healthy | |
|---|---|---|---|---|---|
| | Reference (C) | Alternative (T) | | Reference (C) | Alternative (T) |
| | 0.27 | 0.73 | | 0.47 | 0.53 |
| | | | | Frequency - Psoriasis | |
| | | | | Reference (C) | Alternative (T) |
| | | | | 0.29 | 0.71 |

B

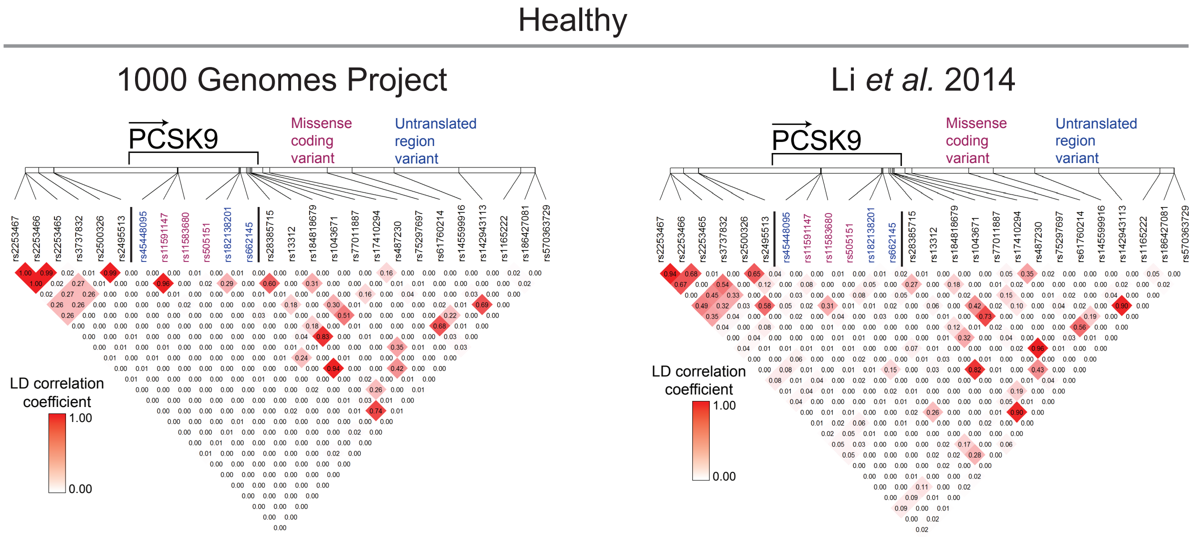


**Supplemental Figure 1. *PCSK9* SNP linkage disequilibrium calculated based on the psoriasis RNA-Seq dataset (12) and 1000 Genomes Project data**. Each square shows a linkage disequilibrium value between two SNPs listed above.

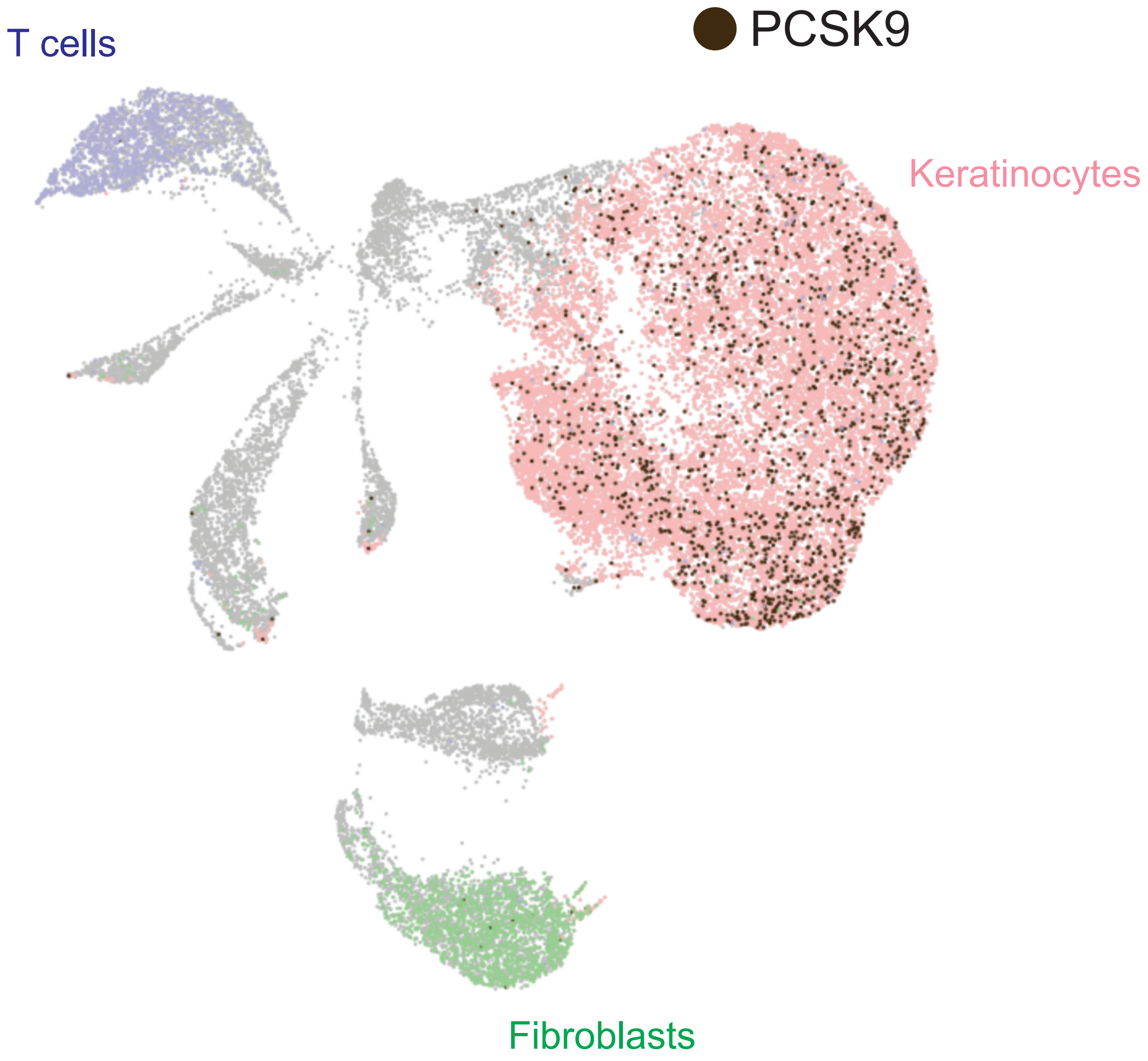


**Supplemental Figure 2. *PCSK9* is mainly expressed by keratinocytes in the skin.** Single-cell sequencing of psoriatic lesional skin (n= 9). Skin biopsies were dissociated and sequencing libraries were generated using a 10X Chromium controller. The UMAP method was then used to create 2-dimensional representation of the resulting data. Keratinocyte, fibroblast and T cell populations were identified by the expression levels of validated markers (*KRT5, KRT14, KRT1* and *KRT10* for keratinocytes, *VIM* and *PDGFRA* for fibroblasts, *CD3* for T cells). *PCSK9*-expressing cells are depicted in dark brown.

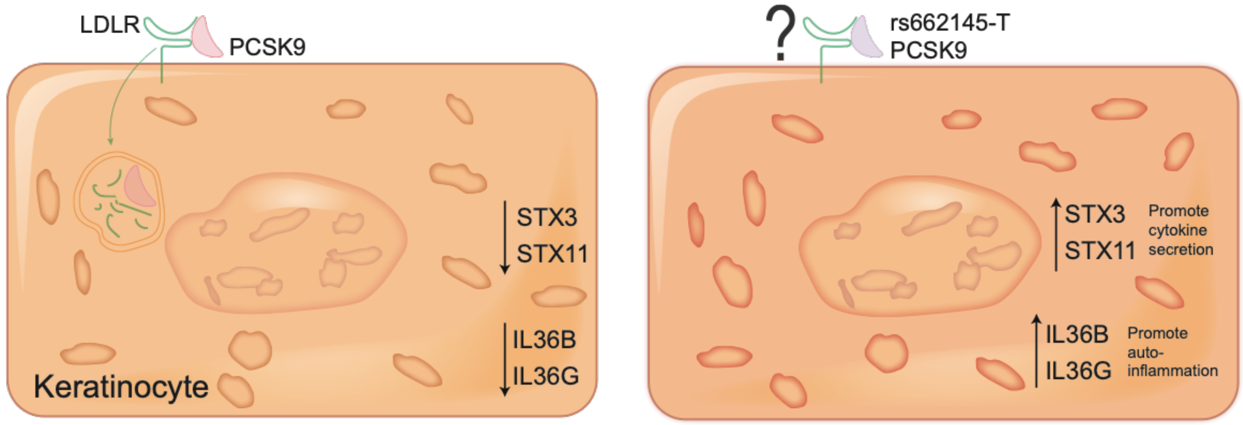


**Supplemental Figure 3. *PCSK9* variant rs662145-T is associated with increased production of autoinflammatory cytokines.** In normal keratinocytes (left), the *PCSK9* protein functions by binding to the transmembrane LDL receptor and initiating vesicle endocytosis of the bound complex followed by intracellular degradation. Normally, individuals have relatively low levels of STX3, STX11, IL-36β, and IL-36γ in their skin. However, *PCSK9* rs662145-T variant individuals (right) have higher expression of IL-36β and IL-36γ in their skin, key psoriasis-associated cytokines.

**Supplemental Table 1. Individual RNA-Seq datasets used in this study**

| Dataset | Sequence Read Archive (SRA) Accession Number | Healthy Samples | Lesional Psoriasis Samples | Uninvolved Psoriasis Samples | Lesional Atopic Dermatitis Samples |
|---|---|---|---|---|---|
| Li *et al.* 2014 (12) | SRP050971, SRP035988 | 90 | 99 | 27 | NA |
| Tsoi *et al.* 2019 (20) | NA | 38 | 28 | 27 | 21 |
